# Review of the Properties of Cement-Based Composites with Carbon-Based Nanomaterials for Potential Functional Applications

**DOI:** 10.3390/ma19112403

**Published:** 2026-06-04

**Authors:** Eryk Goldmann, Marcin Górski, Barbara Klemczak, Rafat Siddique

**Affiliations:** 1 Department of Structural Engineering, Faculty of Civil Engineering, Silesian University of Technology, 44-100 Gliwice, Poland; marcin.gorski@polsl.pl (M.G.); barbara.klemczak@polsl.pl (B.K.); 2 Department of Civil Engineering, Thapar Institute of Engineering & Technology, Patiala 147004, Punjab, India; siddique_66@yahoo.com

**Keywords:** carbon nanomaterials, cement nanocomposites, functional cement materials

## Abstract

The inclusion of carbon nanomaterials in cement-based materials influences a variety of properties, ranging from basic properties to electrical conductivity, which allows for the creation of functional materials. These materials can be utilized as sensors of strain and cracks, as well as to generate heat and harvest electricity. Through the combination of standard applications of construction materials and added functionality, it is possible to create modern construction materials combining higher durability and strength with additional functionality. The enhanced durability of functional cementitious nanocomposites can reduce the need for retrofitting and decrease resource consumption. Together with the increased safety offered by their functional applications, these characteristics make them well aligned with the growing demand for environmentally sustainable construction materials. The presented paper describes the application of various carbon nanomaterials in cement-based composites. Current research directions concerning the influence of the carbon nanomaterial addition on the most important properties of cement pastes, mortars and concretes have been described, along with a critical analysis of the acquired results and further recommendations. Furthermore, recent progress in the development of functional cement-based nanocomposites has also been reviewed.

## 1. Introduction

Nanotechnology is a branch of science widely utilized for modifying material properties on a molecular level. In civil engineering, the addition of nanomaterials in the form of metallic oxides, minerals or carbon nanomaterials is used to improve the mechanical and functional properties of construction materials. Besides an improved mechanical strength, they are characterized by higher corrosion resistance; self-cleaning capabilities; hydrophobic layers for monument protection; and additional functionality, including self-sensing, heat generation, energy harvesting and self-healing. These functional properties allow for the use of construction materials beyond their inherent structural role. Changes in microstructure and the addition of functional properties to construction materials might lead to a reduction in their environmental impact through a reduction in resource consumption, retrofitting needs and the improvement of users’ safety. This allows the construction sector to fit into the assumptions of the UN Sustainable Development Goals [1] and New European Bauhaus [2].

Although the present review focuses on the functional applications of cement-based nanocomposites, understanding the influence of carbon nanomaterials on fundamental material properties remains essential. Properties such as rheology, hydration kinetics, shrinkage development and microstructure directly affect nanomaterial dispersion, conductive network formation, durability and long-term reliability of functional behaviour. Therefore, discussion of these properties is intended not as a general description of cement composite behaviour, but as a mechanistic basis for understanding the performance of multifunctional cementitious systems. To ensure a structured and comprehensive assessment of the available research, the reviewed literature was selected based on relevance to carbon-based nanomaterials and their influence on both the fundamental and functional properties of cement composites. The literature survey included studies focused on carbon nanotubes, graphene-based materials and carbon nanofibres in cementitious systems, with particular emphasis on rheological behaviour; hydration; microstructure; mechanical performance; and multifunctional applications such as self-sensing, self-heating and energy harvesting. Keywords including ‘carbon nanomaterials’, ‘cement composites’, ‘carbon nanotubes’, ‘graphene’, ‘self-sensing’, ‘electrical conductivity’, ‘energy harvesting’ and related terms were considered during the literature search. Preference was given to peer-reviewed studies and highly cited and recent publications directly related to functional cementitious materials, while studies outside the scope of cement-based applications or lacking sufficient experimental relevance were excluded. The collected findings were synthesized through comparative analysis of reported mechanisms, material performance and application perspectives.

Besides conventionally investigated carbon nanomaterials such as carbon nanotubes, graphene and carbon nanofibres, recent studies have identified emerging nanoscale carbon materials, including carbon quantum dots (CQDs) and graphene quantum dots (GQDs), as a promising research direction in cementitious composites. Due to their nanoscale dimensions, high specific surface area and unique surface chemistry, these materials may influence the hydration processes, microstructural development and multifunctional properties of cement-based materials. Recent investigations demonstrated improvements in the mechanical performance, durability and dynamic behaviour of concrete through the use of graphene quantum dots and related nanostructures [3]. Furthermore, conductive cement composites incorporating GQDs and supra-GQD assemblies showed enhanced electrical performance and potential for multifunctional applications [4]. Carbon quantum dots were also reported to positively influence the microstructural development and mechanical properties of cement mortars [5]. Recent review studies indicate rapidly increasing research interest in CQD applications in construction materials and identify them as a potentially important future research direction [6]. However, despite their promising characteristics, research concerning CQD- and GQD-based cement composites remains at an early stage. Therefore, the present review focuses primarily on conventionally studied carbon nanomaterials currently dominating research on functional cement composites.

Despite the large number of studies dedicated separately to the carbon nanomaterial modification of cementitious composites and to functional cement-based materials, available review papers frequently focus either on individual nanomaterials or on isolated functional applications. A comprehensive discussion linking the influence of carbon nanomaterials on fundamental material behaviour with their role in determining multifunctional performance remains limited. Therefore, the original contribution of the present review is the integration of these two perspectives through a mechanism-oriented analysis connecting material-scale modifications with engineering functionality. Particular emphasis is placed on relationships between nanomaterial dispersion, rheological and hydration behaviour; microstructure development; and conductive network formation as governing factors controlling self-sensing, self-heating and energy harvesting performance. In addition, comparative evaluation of different carbon nanomaterials, including their advantages, limitations and practical implementation challenges, is provided to identify current knowledge gaps and future research directions.

**Figure 1:**
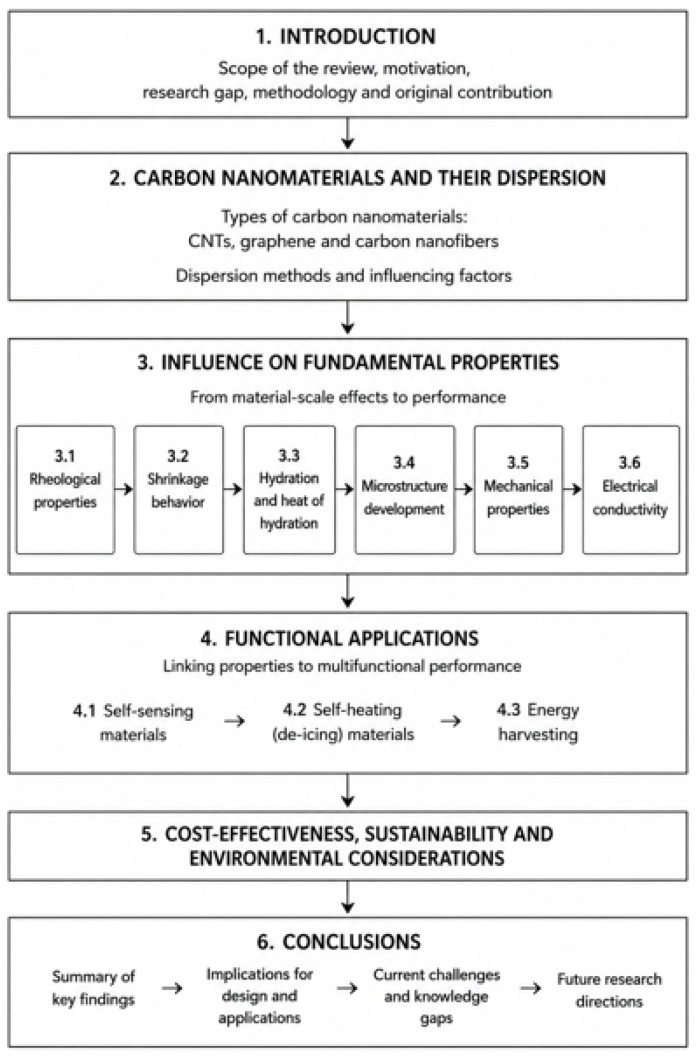
Flowchart of the review’s structure.

To improve readability and provide a clearer overview of the manuscript organization, the structure of the present review follows a progressive approach linking material characteristics with functional applications. The review begins with a discussion of carbon nanomaterials and their dispersion methods, since dispersion quality represents one of the key factors governing nanocomposite performance. Subsequently, the influence of carbon nanomaterials on the fundamental properties of cementitious composites, including rheology, shrinkage, hydration, microstructure, mechanical behaviour and electrical conductivity, is analysed. These material-scale mechanisms are then linked with functional applications of cement-based nanocomposites, particularly self-sensing systems, self-heating materials and energy harvesting technologies. Finally, practical implementation aspects, sustainability considerations, current knowledge gaps and future research directions are discussed. A schematic flowchart illustrating the logical progression and structure of the review is provided in Figure 1 to facilitate navigation through the discussed topics.

## 2. Carbon Nanomaterials and Their Dispersion

Carbon nanomaterials are synthetic allotropes of carbon and are widely used in multiple branches of modern industry, from electronics and energetics, medicine and environmental engineering to materials engineering. Arranged structures of one, two and three dimensions of carbon atoms allow for the creation of materials with unique mechanical and conductive properties, while the ability of carbon to merge with other elements opens a possibility of functionalizing carbon nanomaterials for specific use. In construction materials, the most used carbon materials are one-dimensional carbon nanotubes and nanofibres and two-dimensional forms of graphene.

### 2.1. Carbon Nanotubes

**Figure 2:**
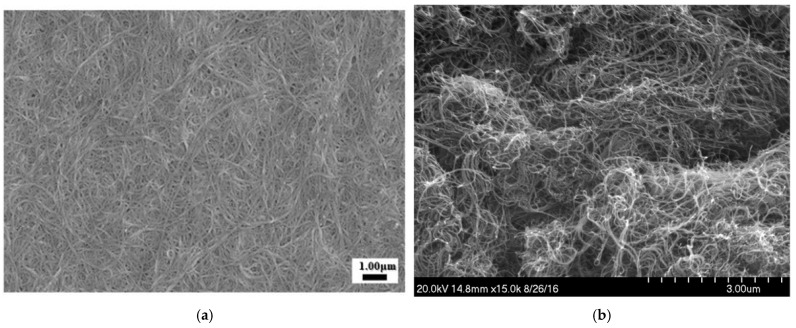
Microscopic images of carbon nanotube types: (**a**) single-walled nanotubes [13]; (**b**) multiwalled nanotubes [14].

Carbon nanotubes (CNTs), which are one-dimensional elongated carbon nanomaterials, were first described by Japanese scientist Sumio Iijima in 1991 [7]. They are a form of a rolled graphene sheet with halves of fullerene at the ends and are divided into two main groups: single-walled carbon nanotubes (SWCNTs) and multiwalled carbon nanotubes (MWCNTs). The diameter of single-walled carbon nanotubes is usually between 1 and 2 nm and multiwalled between 2 and 100 nm [8]. Because of their elongated structure, carbon nanotubes have a very high electrical conductivity of 10^6^ S/m for SWCNTs and 10^5^ S/m for MWCNTs [9] and excellent mechanical properties. Tensile strength is reported in a range of 500 GPa [10] for single-walled nanotubes and 10–63 GPa [11] for multiwalled nanotubes, while their Young modulus is estimated as 1.0 TPa [12]. Example microscopic images of both types of carbon nanotubes are presented in Figure 2.

### 2.2. Graphene

**Figure 3:**
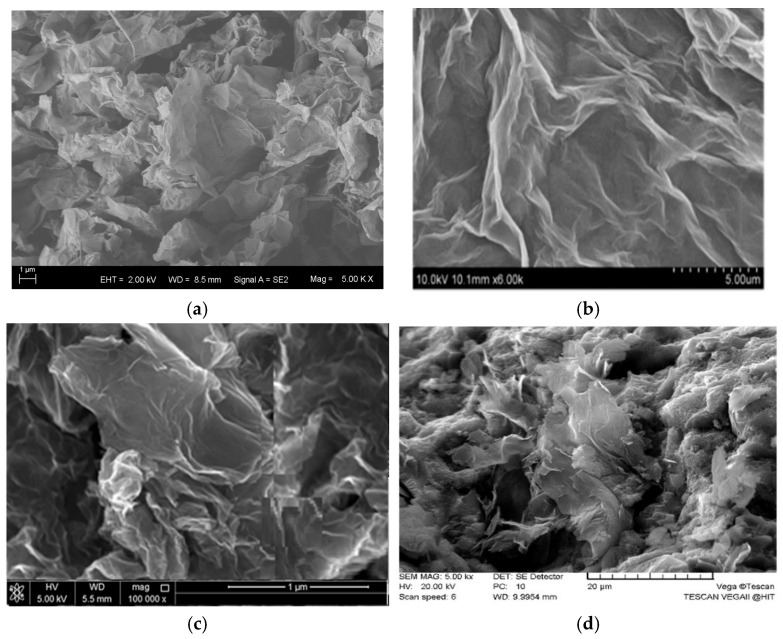
Microscopic images of various forms of graphene: (**a**) pure graphene [19]; (**b**) graphene oxide (GO) [20]; (**c**) reduced graphene oxide (rGO) [21]; (**d**) GNP [22].

Graphene, a two-dimensional carbon nanomaterial, was described by Novoselov and Geim in 2004 when they acquired pure graphene flakes using mechanical exfoliation of graphite [15]. Graphene can be acquired in pure form, as graphene oxide (GO), reduced graphene oxide (rGO) and interconnected layers called graphene nanoplatelets (GNP). All forms of graphene are shaped in a honeycomb lattice with carbon atoms arranged in hexagons to which, in the case of GO and rGO, functional groups are attached [16]. Example microscopic images of various forms of graphene are given in Figure 3. Graphene forms are different in terms of material properties, especially electrical conductivity and ease of dispersion in water. Functional groups of graphene oxide increase its dispersibility in water but reduce the electrical conductivity related to the pure material, while reduced graphene oxide is considered to be an intermediate form that combines the properties of both graphene oxide and the pure form. Similarly, as with carbon nanotubes, the tensile strength of graphene is very high, around 130 GPa, with a Young’s modulus in the range of 1 TPa [17]. The electrical properties of graphene, described with electron mobility, are 200,000 cm^2^ V^−1^ s^−1^ [18].

### 2.3. Carbon Nanofibres

**Figure 4:**
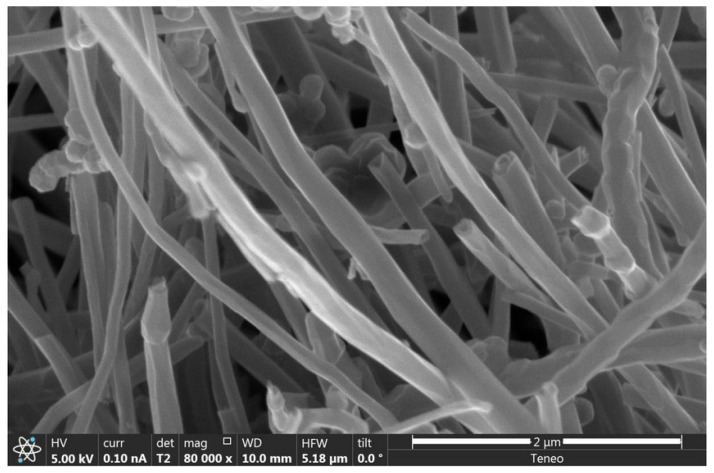
Microscopic image of carbon nanofibres [26]. An image of only one type is presented due to the minimal visual differences between CNF types.

Carbon nanofibres (CNF), similar to carbon nanotubes, are classified as one-dimensional materials. They are composed of layers of graphene stacked along the fibres’ axis. The following types can be distinguished depending on the angle between the layers and the axis [23]: platelet (90° angle), tubular (0° angle) and fishbone (angle between 0° and 90°) [24]. A microscopic image of carbon nanofibres is given in Figure 4. The mean length of carbon nanofibres is usually 10 µm, and their diameter is 50–200 nm [25]. The tensile strength of carbon nanofibres is close to 7 GPa, and Young’s modulus is between 0.4 and 0.6 TPa [10].

### 2.4. Water Dispersion

Water dispersion of carbon nanomaterials is a crucial and widely discussed topic amongst researchers dealing with cement-based nanocomposites [27,28,29,30,31,32,33,34,35,36,37,38,39,40,41,42,43,44,45,46,47,48,49,50,51]. A wide variety of techniques for aiding and characterizing the water dispersion are analysed and devised, including the use of additives to aid the homogeneity of water dispersion, as well as different mixing procedures, including ultrasonic mixing.

The use of additives is usually considered a safe way to add water dispersion since it does not physically damage the nanomaterial structure, unlike mixing. Typically, one of the two types of surfactants is used to aid dispersion: surfactants that are usually used in cleaning products or plasticizers used in concrete chemistry. Depending on the main mechanism, surfactants can be categorized as ionic, anionic or amphoteric, amongst which, for cementitious nanocomposites, SDS, SDBS, CTAB, Triton X100, Tween 20 and Brij 35 are utilized [35,45,46,49,52,53,54]. Carboxymethyl cellulose and gum Arabic are other examples of additives commonly used in the food industry but also used for aiding the water dispersion of carbon nanomaterials [29,35,49]. Amongst superplasticizers and concrete additives used for cement nanocomposites, a majority of literature research suggests the polycarboxylate type [28,30,31,32,36,43,44,47,51,55]; however, there are also examples of using naphthalene-based plasticizers [30,37]. The main advantage of using concrete additives is their well-known influence on the properties of cement-based composites, while surfactants might have negative side effects, including an increase in porosity due to large bubble generation.

Sonication is a process of mixing that utilizes high-frequency vibrations to convey energy to the suspension, causing nanomaterial agglomerations to split. It is usually used in conjunction with the previously discussed additives to ensure a homogeneous distribution of nanomaterial particles in the water suspension. It can, however, damage nanomaterials if the sonication time is too long [36,43,55]. Sonication parameters are often described using either the total time or the total energy conveyed to the suspension. Due to variations in nanomaterial types and the preparation regimes of water suspensions, large discrepancies can be found in the literature concerning the optimal time, which ranges from 10 min [41] to 5 h [52], while optimal total energy is considered to be between 25,000 J [47] and 170,000 J [43].

Proper dispersion of nanomaterials is crucial in order to avoid large agglomerations, which can mitigate the positive influence of nanomaterial addition on the properties of cementitious composites. A non-homogeneous distribution of nanomaterial particles in water suspensions, which is the most common way of introducing carbon nanomaterials into the cement matrix, can cause similar issues in the matrix itself. Local agglomerations can impair strengthening mechanisms and cause discontinuity in the conductive paths required to create functional materials.

With a large variety in carbon nanomaterial structures, quality and behaviour, it is difficult to provide a universal solution for proper water dispersion. Different techniques suitable for specific types of nanomaterials might not be enough for other batches of even the same type; therefore, establishing individual processes for each case is recommended. The general consensus is that the use of additives combined with mixing, usually ultrasonic mixing, is the most effective way to improve the homogeneity of water dispersion of carbon nanomaterials, with specific parameters and types of additives selected depending on the nanomaterial used.

## 3. Properties of Cement-Based Composites

The addition of carbon nanomaterials influences a variety of properties of cement-based composites. Due to a small size, a large surface area and hydrophobic properties, even a small dosage can influence the basic and electrical properties of cement-based materials. Interactions between carbon nanomaterials and cement, aggregates, mixing water and chemical additives are complex and, in many cases, not yet fully understood. The following sections describe the research results of tests of the basic properties of cement-based materials with the addition of carbon nanomaterials. Available research conducted on cement pastes as the most basic type of material, but also mortars and concretes, was considered to determine the important influence of aggregates. The inclusion of various types, dosages and combinations of carbon nanomaterials, as well as combining them with larger-scale fibres, demonstrates a multidimensional approach and a wide interest in the topic of cement-based nanocomposites.

### 3.1. Rheological Properties of Fresh Mix

The consistency and fluidity of a fresh mix are often described either using a simplified method through its flow or a more precise rheological parameter measurement. These parameters describe yield stress, which is the minimal shearing stress required to initiate the flow of a viscous–plastic material and plastic viscosity, which describes the value of shear stress needed to keep the flow under a specified shearing speed. Properties describing the consistency of cement-based materials determine their practical usability and ease of work regarding the casting of elements and application. The main factors influencing the rheological properties of cement-based composites are their composition, especially the water-to-cement ratio and the addition of chemical or mineral compounds. A summary of selected results concerning the rheological properties of cement composites with the addition of carbon nanomaterials is given in Table 1.

The addition of carbon nanomaterials mostly leads to a reduction in the flowability of cement-based materials, with a reduction in flow radius and an increase in yield stress and plastic viscosity [62]. This phenomenon is attributable to complex interactions between nanomaterial particles, mixing water, cement grains and water-reducing agents [56]. Adsorption of water and water-reducing additions on the surface of carbon nanomaterials can lead to a reduction in water available to wet cement grains and reduce the efficiency of superplasticizers. An important factor in this case is the proper dispersion of the nanomaterial in the cement matrix. Agglomerations created by improper dispersion can adsorb more water and plasticizers, which leads to further reductions in their efficiency. 

In cases of one-dimensional materials, an additional effect of entanglement might be possible, which can enlarge the agglomerations and connect them. This effect was observed by Jiang et al. [57]; however, the addition of carbon nanofibres did not significantly impact the yield stress of cementitious composites. An indirect effect of lowering the flowability of cement-based materials with carbon nano additions can be their influence on the hydration reaction through the nucleation effect, which causes faster setting of cement pastes and, therefore, a reduction in their flow [58,63].

Available research points out the possibility of using standard additions used in construction materials engineering to reduce the negative influence of carbon nanomaterials on the flowability of cement-based materials. Fly ash [59] and granulated blast furnace slag [60] are commonly used supplementary cementitious materials that have been proven to reduce the yield stress of cement pastes with graphene and GO. An emphasis was also put on the sensitivity of rheological properties with regard to small changes in the water-to-cement ratio [63]. In a study by Jiang et al. [57], conducted on cement pastes with the addition of carbon nanofibres and with a very low water-to-cement ratio, a reduction in yield stress of 80% was achieved when the water-to-cement ratio increased from 0.20 to 0.22. Another proposition given in [61] was a surface modification of GO by the application of a nano-silica layer, which significantly reduced the yield stress and plastic viscosity of cement pastes at the same dosage of nanomaterial. A specially engineered suspension of dispersed CNTs, prepared for large-scale applications, reduced the influence of the nanomaterial on the flowability of concrete mixes, as presented in [63]. The addition not only improved the dispersion of CNTs but also reduced the adsorption effect of water and the superplasticizer and directly improved the flowability of tested mixes. Besides direct water availability, the effect of the water-to-cement ratio may also be linked with competitive adsorption phenomena occurring among cement grains, nanomaterial particles and chemical admixtures. Carbon nanomaterials possess a very high specific surface area and can adsorb both water molecules and superplasticizer molecules on their surfaces, reducing the amount of dispersant available for cement particle separation and decreasing the steric repulsion effect responsible for maintaining flowability [38,64]. This phenomenon may significantly reduce the efficiency of superplasticizers despite maintaining a constant dosage. Furthermore, previous investigations demonstrated that the influence of superplasticizers used during nanomaterial dispersion may be comparable to, or even greater than, the direct influence of carbon nanomaterials themselves on hydration and fresh-state behaviour [65]. Nanomaterial agglomerates may additionally trap water within their structure and alter local hydration conditions, further reducing the amount of free water available in the system [66]. Consequently, relatively small changes in the water-to-cement ratio may affect not only the amount of available water but also alter adsorption equilibrium and dispersing efficiency. Therefore, the rheological properties of cement-based nanocomposites should be interpreted as the result of coupled interactions between water, nanomaterials and chemical admixtures rather than solely as a direct consequence of water content.

The literature indicates a negative influence of the addition of carbon nanomaterials on the rheological properties of cement-based composites. Complex mechanisms, including water and water-reducing agent adsorption due to the large surface area of nanomaterials and the possible influence on setting time, are listed as the main causes of reduced flowability. Further and more detailed tests of the scale of influence of each of these mechanisms could improve the overall understanding of the influence of carbon nanomaterials on the rheological behaviour of cement-based composites and improve the effectiveness of counteracting them. Regardless, it is possible to effectively reduce the negative influence of the described mechanisms using typical additions and composition modifications used in building materials engineering.

### 3.2. Shrinkage

Shrinkage is a rheological phenomenon that occurs in hardened cementitious materials. It is a sum of strains caused by the loss of water and chemical reactions in the cement matrix. The intensity of shrinkage depends on the mineral composition of the matrix, the proportions and types of the composite components, and conditions during setting.

Research available in the literature considering the influence of carbon nanomaterials on the shrinkage of cement-based composites is not in agreement. Some of this research shows an increase [67,68,69,70,71,72,73,74,75,76,77] in shrinkage strains, while some shows a decrease [66,78,79,80]. There is no clear distinction in the influence on shrinkage strains between cement pastes, mortars and concretes. The main mechanism is still dependent on the shrinkage of the cement matrix itself, and in the case of all tested materials, it was linked with the dosage of the nanomaterial. Similar interactions between nanomaterials and matrix constituents, mainly water and additives, occur in all types of materials, which suggests that aggregate confinement does not influence the shrinkage of cement nanocomposites in a decisive manner. Table 2 compares the selected results of shrinkage tests from the analysed literature.

One of the reasons for a reduction in shrinkage strains is a reduction in the porosity and densification of the cement matrix [72,73,76,77]. This effect is caused by the intensification of the hydration reaction and an increase in the hydration rate, which leads to the creation of a higher number of reaction products, which densify the microstructure. Another possible explanation is the effect of physically filling the pores usually occupied by water. An example of shrinkage strain reduction caused by the addition of carbon nanomaterials was described in [72], where the addition of 0.05 wt.% CNT reduced the shrinkage strain of cement mortars by 9%. The reduction was explained by the filling of pores filled with water with carbon nanotubes instead. A reduction in the porosity of the cement matrix in the range of the smallest pore diameter leads to a reduction in capillary porosity and the pressure in them [71,76], which reduces the shrinkage strain caused by self-draining.

An additional influence of carbon nanotube agglomerates that was pointed out in [71] is that they might trap water in the structure of the agglomerate. This effect, as evident in the mentioned research, reduced the humidity in the first stage of hydration reaction; however, it also reduced the autogenous shrinkage of cement pastes in a long-term test. A similar mechanism was observed for concretes with graphene oxide addition [78]. In the first 3 days, the addition of nanomaterial increased the shrinkage strain; however, from the perspective of 60 days, the addition of GO reduced the rate of shrinkage increment, which was caused by an initial absorption of water and the gradual release of it with time. This self-nourishing effect reduced the drying shrinkage over a longer time period.

Most literature research points out a threshold for the nanomaterial dosage, which has a positive influence on shrinkage strains. Too-low dosages might have a negligible influence, while too-high dosages cause problems with proper dispersion, which negates the positive effect. This positive effect on shrinkage strains was mostly observed for relatively low dosages of carbon nanomaterials, mainly: 0.05% to 0.1% for CNT [71,72,73,74], 0.01 wt.% for CNF [77], and values between 0.05 wt.% [67] and 0.3 wt.% for GO [68].

The issue of shrinkage is an important consideration for most concrete and reinforced concrete structures due to crack propagation, which can pose a serious threat to the usability and durability of a structure. Available research shows, in most cases, the positive influence of the carbon nanomaterial addition on the shrinkage of cement-based composites under proper dosage. This influence is mostly caused by a reduction in porosity through the filler effect and a possible influence on hydration mechanics and the gradual release of water trapped in nanomaterial agglomerations. Differences in research results concerning cement pastes and concretes require a deeper analysis and tests in order to clearly define the influence of the presence of aggregates on the shrinkage strain reduction caused by the addition of carbon nanomaterials.

### 3.3. Heat of Hydration

The hydration reaction is the fundamental reaction that takes place during the setting of cement-based materials. It is strongly exothermic, which allows for the precise testing of its progress using calorimetric methods. Differences in heat released and the rate of release allow for an assessment of the influence of various additions and composition modifications on changes during cement setting. The influence on the hydration reaction is mainly attributed to the chemical composition of cement, especially the content of C_2_S and C_3_S and the presence of mineral additions and chemical admixtures. The course of the hydration reaction is described based on typical phases related to the intensity of heat release during the reaction of specific cement components. The following phases can be distinguished:

- The initiation phase, in which a rapid heat release takes place, along with easily soluble ions.
- The induction phase: Inhibition of the hydration reaction and a decline in heat release to values close to zero.
- Acceleration phase: The beginning of hydration of tricalcium silicates (C_3_S) and an increase in heat release. The second peak of the heat release curve is located in this phase.
- Deceleration phase: The end of the hydration of silicates and a gradual reduction in heat release.
- Decay phase: The slow continued reaction of remaining minerals. The heat release curve asymptotically approaches zero.

The influence of carbon nanomaterials on the heat of hydration during the setting of cement-based composites is unclear. There are examples of studies in the literature that report not only a possible acceleration in the reaction and intensification of heat release [58,68,80,81,82,83,84,85,86,87,88] but also its deceleration [48,80,89,90]. Other sources claim that carbon nanomaterials have an infinitesimal influence on hydration heat release [47,66,91,92,93]. The main reason for the intensification of the reaction can be the nucleation effect, which causes hydration products to grow around nanomaterial particles. Another possible explanation is the bridging of cement grains by nanomaterials, which could intensify the reaction [77]. Among possible factors that slow down hydration heat release, the most commonly indicated mechanisms include: physical separation of cement grains by nanomaterials; water adsorption, which reduces the amount of water available for hydration; and the physical covering of cement grains by nanomaterial agglomerations [48], which hinders soluble ion release in the early phase of reaction.

Intensification of hydration heat release in the main peak was observed for cement pastes with graphene oxide in [58]. A possible reaction between functional groups and hydration products, which intensified the nucleation effect, was pointed out as the main cause of this phenomenon. Similar results were obtained by Xu [68], who observed an intensification in heat release in the early phase of hydration for samples with 0.1 wt.% and 0.3 wt.% GO by around 20%. The effect in the final stage of the measurements was negligible. Again, the main reason for the effect was assumed to be connected with the nucleation effect, which was stronger for GO due to electrostatic attraction caused by the functional group. A similar influence for CNT was observed in [85], where the addition of nanomaterial clearly intensified the hydration reaction in the induction phase but diminished with time. A significant influence on hydration heat release in the first three phases was observed by Liu for a hybrid material that combined carbon and titanium nanotubes [86]. The combined influence of CNT and superplasticizer was tested by MacLeod in [65]. An increase of 45% in the rate of hydration heat release was observed in the first stages of the reaction, and it grew for larger dosages of CNT; however, in the later stage, the influence of the superplasticizer proved to be greater. On the other hand, results acquired by the authors of [90] showed a delay in the main peak with an increasing dosage of carbon nanotubes, while the influence of sonication time, and with it, the quality of dispersion, was minor. The research of Meng and Khyat [80] compared the mutual influence of the addition of graphene platelets and carbon nanofibres on the hydration heat generation of ultrahigh-performance concrete. The calorimeter curve was observed to have a different shape for each of the nanomaterial additions. In the case of CNF, a prolongation of the induction period of 5% was observed, and the cumulative heat was lowered by 35%, which was possibly caused by CNFs covering cement grains and the larger dosage of superplasticizer required for proper flowability in the mix. For GNPs, the induction period started 50% earlier than that of the reference samples, and the cumulative heat was increased by 45% after 72 h, which could have been caused by the strong nucleation effect of nanomaterial with a large surface area. A negligible effect for different types of carbon nanomaterials was observed in [87]. The addition of GNP, GO and CNT, in both standard and functionalized forms, increased the rate of heat released in the main peak and cumulative heat released for cement pastes; however, the differences were too small. The cause of this result was not deeply discussed. The effect of both carbon nanomaterial and concrete additives, such as metakaolin [91] and silica fume [66], on hydration heat evolution was also tested; however, in both of these cases, the influence of the additive was significantly stronger than that of the nanomaterials themselves.

Although the available literature reports apparently contradictory conclusions regarding the influence of carbon nanomaterials on hydration heat evolution, these differences can largely be explained by the coexistence of competing mechanisms and by variations in experimental procedures. Carbon nanomaterials may simultaneously accelerate and retard hydration depending on which mechanism becomes dominant under specific conditions. The acceleration effect is mainly associated with the nucleation mechanism, where nanomaterial particles provide additional surfaces for the growth of hydration products. Their high specific surface area promotes early C-S-H formation and may shorten the induction period [65]. This effect can be particularly pronounced in graphene oxide due to the presence of oxygen-containing functional groups that enhance interactions with cement hydration products and electrostatic attraction mechanisms [58]. In contrast, several physical mechanisms may delay hydration kinetics. Carbon nanomaterials can adsorb water within their agglomerated structures, reducing the amount of free water available for hydration [92]. Agglomerates may also physically cover cement grains and hinder ion dissolution during the early stages of hydration [48]. Furthermore, poor dispersion quality can intensify these effects because larger agglomerates create localized regions where hydration products develop less efficiently. Previous studies on MWCNT-modified cementitious composites additionally showed that prolonged sonication or specific combinations of nanotubes and superplasticizers may significantly delay the occurrence of the main hydration peak and reduce early heat evolution [90]. Such observations indicate that the influence of dispersion procedures and chemical admixtures can be comparable to, or even greater than, the effect of nanomaterials themselves. An additional source of discrepancy is the interaction between nanomaterials and admixtures used to improve dispersion. Superplasticizers, surfactants, and sonication procedures alter hydration kinetics independently of the nanomaterial itself. Previous investigations demonstrated that superplasticizers frequently delay early hydration due to steric effects, while carbon nanotubes may accelerate or retard the process depending on dosage and dispersion conditions [65]. Therefore, the observed calorimetric response often reflects the combined action of nanomaterials and dispersion-related additives rather than the isolated effect of the nanomaterial. Consequently, contradictory literature findings should not be interpreted as mutually exclusive results but rather as outcomes of a balance between competing acceleration and retardation mechanisms. In many cases, nanomaterials affect only selected hydration stages, especially the induction and acceleration periods, while their influence on cumulative heat release at later ages remains limited.

Considering all of the analysed literature research, the influence of carbon nanomaterial addition on the hydration heat of cement-based composites is still in question. Results showing increases, decreases and negligible effects on hydration heat evolution and cumulative heat can be found. Moreover, usually, only one of the phases of the hydration reaction is affected by these changes. Most of the time, mechanisms involved in the influence on hydration heat evolution include the nucleation effect; the filler effect for intensification and water adsorption; and the covering of cement grains for a decrease in intensity. Moreover, the influence of superplasticizers used for both fluidity and to aid water dispersion is known, but their potentially complex interaction with carbon nanomaterials might introduce a different effect on the hydration heat evolution than just the superplasticizer. The determination of the influence of both carbon nanomaterials and superplasticizers alone and in different combinations on hydration heat evolution. and especially the scale of influence for nanomaterials, could prove to be key to resolving this issue for future practical applications.

### 3.4. Microstructure

The microstructure of the cement matrix is composed of hydration products that form a series of connections between minerals during setting. The majority of the microstructure consists of amorphous calcium silicate phase (C-S-H), calcium hydroxide (Portlandite), and various crystalline forms of ferro aluminates, mainly Ettringite and other minerals created from impurities in cement. Cement matrix is naturally porous due to the consumption and evaporation of mixing water, entrapment of air bubbles during mixing and the addition of air-entraining admixtures. Pores create a complex network in the matrix, which includes open, closed and semi-open pores of different diameters. As defined by the International Union of Pure and Applied Chemistry (IUPAC) with regard to size, pores can be classified as micropores (<2 nm), mesopores (2–50 nm) and macropores (>50 nm).

Examination of the cement matrix’s microstructure is usually done through quantitative methods, which allow for the identification of mineral structure and the calculation of hydration degree and porosity. The most popular methods used include thermogravimetric analysis (TGA), X-ray diffraction (XRD), and porosimetry methods, mainly mercury intrusion (MIP) and microcomputed tomography (micro-CT). Additionally, micromechanical tests are employed to assess the hardness and mechanical properties of identified phases, and microscopy methods are used to visually assess the shape of identified phases.

The influence of carbon nanomaterials on the microstructure and porosity of the cement matrix is usually attributed to two main phenomena. The nucleation effect, which causes hydration products to grow around nanomaterial particles, promotes the growth of closely packed minerals and densification of the matrix [77,94]. The second mechanism is the filler effect of nanomaterial particles and agglomerations physically filling the smallest pore sizes and refining the pore structure [95], causing the porosity curve to shift towards bigger diameters. Differences in the crystal orientation of Portlandite [96] and Ettringite [88] have also been observed for cementitious composites with the addition of carbon nanomaterials.

A proper dispersion of carbon nanomaterials in the cement matrix was considered a crucial factor for microstructure refinement in [97], where a significant reduction in hardness was measured around CNF agglomerations, while the amount of high-stiffness C-S-H was reduced. The importance of proper dispersion of graphene flakes was emphasized by Du and Pang in [31], who noticed a 37% reduction in total porosity and a 30% reduction in average pore diameter in cement pastes with 1.0 wt.% of well-dispersed nanomaterial. In research by Gao [32], the addition of 0.2 g of GO and 0.4 g of CNT reduced the porosity of cement pastes by 6.5%. A significant densification of the microstructure of cement paste with 0.1 wt.% CNT was observed by MacLeod [94], who attributed this result to the nucleation effect being much more pronounced with well-dispersed nanomaterials. The importance of an optimal nanomaterial dosage was highlighted by Chen and Akono [98], in whose research dosages of carbon nanotubes lower than 0.5 wt.% reduced the porosity of cementitious composites, especially for small pore diameters; however, a dosage of 1.0 wt.% increased total porosity by 79% compared to a reference sample. An important comparison was made in [95], where various diameters of carbon nanotubes were compared. As a result, nanomaterials with smaller diameters were much more effective in reducing the porosity of the cement matrix due to a more pronounced filler effect. Qureshi [99] described a positive effect of multiple forms of graphene on the microstructure of cement paste, which was caused by the promotion of C-S-H and Portlandite growth in samples owing to the addition of graphene. This effect reduced total porosity by 2.6%. For graphene oxide, results from Yu [100] and Chu [101] showed reductions in the porosity of ultrahigh performance concrete with 0.04 wt.% and 0.05 wt.% GO respectively. The effect of carbon nanofibres connecting hydration products and, therefore, a reduction in the porosity of cement pastes by 2.92% with 0.1 wt.% CNF was observed by Akono [102].

The influence of carbon nanomaterial on the microstructure characteristics of cement-based composites is generally considered to be positive under the assumption of proper dispersion and optimal dosage. The main mechanisms contributing to the improvement of microstructure properties and reduction in porosity are connected to hydration reaction promotion, especially phases with high density and stiffness, and the mechanism of physical filling of pores with nanomaterial particles. The results of detailed analyses using advanced techniques reveal the influence of nanomaterial mineral and phase composition of the cement matrix, as well as its porosity and micromechanical properties; however, due to the cement matrix being nonhomogeneous, these results are valid for a small area of the entire volume of the composite and allow for averaging these properties for the entire material. Further research should focus on a more precise understanding of the physical interaction between nanomaterials and hydration products, as well as the theoretical and numerical modelling of the enhanced microstructure.

### 3.5. Mechanical Strength

Mechanical strength is one of the most important properties of cement-based materials, which determines their practical usage. Usually, the compressive strength is the main factor considered in the design of concrete and reinforced concrete elements due to their working conditions, while tensile strength is more important when considering crack resistance and durability of the structure. The mechanical strength of cement-based composites is mainly determined by their composition and properties of each component, especially cement and aggregate; however, the water-to-cement ratio, admixtures and additions can also influence their mechanical performance. In the case of reinforced concrete, there is also an additional factor of the bonding interface between the cement matrix and rebars or fibres.

The very high mechanical strength of carbon nanomaterials has little direct influence on the strength of cement-based nanocomposites, mainly due to the difference in scale of such additions compared to longer fibres. Their influence is evident more through the refinement of microstructure and reduced porosity. Elongated nanomaterials like the carbon nanotubes of nanofibres can, to some degree, bridge cracks in the microstructure, which was observed for CNTs in [103] for mortars that had a less brittle failure mode compared to samples without nano addition. A positive influence of carbon nanomaterial addition on the interface zone between fibre reinforcement and the cement matrix was observed for the addition of GO and steel fibres [101] and CNF with polyethylene fibres [104].

Again, many studies highlight proper nanomaterial dispersion in the cement matrix as one of the essential factors in determining their positive influence on mechanical strength [36,38,48,49,105]. Moreover, the dosage should not exceed threshold values specific to the nanomaterial type; otherwise, the resulting agglomerations can lower the mechanical strength of the composite [58,98,106]. In order to show the significant results of research focusing on the mechanical strength of cement-based nanocomposites, only select results are given in Table 3. It is important to note that due to the fundamental importance of mechanical strength, it is often tested even if another property is the main focus of a given study.

In the presented examples, tensile strength was more affected by the addition of carbon nanomaterials than compressive strength. Optimal dosages proposed by the authors of these tests are relatively small, which can be an important factor when considering the economic aspect of scaling the production of cement nanocomposites above the laboratory scale. Small dosages might also be easier to disperse, reducing the influence of dispersion quality and making it easier to produce nanocomposites.

Considering the water-to-cement ratio of the tested composites, most of the discussed literature used ratios close to 0.5, which is often considered in standards as a baseline value for strength tests of cementitious materials. This approach could be considered safe due to standard recommendations, therefore lowering the impact of different w/c ratios. Among studies that considered a water-to-cement ratio between 0.4 and 0.55, the increase in compressive strength falls mostly in the range of 10–30%, with one exception in [48]. Combined with an explanation for the increase in compressive strength being similar in most cases, i.e., the densification of the matrix and a reduction in porosity, it can be concluded that most carbon nanomaterials synergize well with w/c ratios recommended by standards. Lower ratios described in the presented examples were mostly used for concrete mixes, which tend to have precisely tailored compositions to meet the requirements of ultrahigh strength or various other properties, such as self-compacting. Although limited, the results for concrete samples with low w/c ratios demonstrate that carbon nanomaterials can also contribute significantly to the improvement of compressive strength of materials specifically designed for high strength, and their properties do not impair concrete strength. On the other hand, the flexural strength and tensile splitting strength of the presented results show not only higher improvements of up to 120% but also a much larger spread of results, from 26% to 120%. Therefore, there is no clear correlation between the rate of improvement and the water-to-cement ratio used in the presented studies.

Looking at the optimal dosage of the nanomaterial as claimed by these authors, for most of the analysed studies, it does not exceed 0.15 wt.%, with the exception of two of the studies [33,107]. For the lower range of dosages, it can be seen that in most cases, the smaller the dose, the higher the improvement in mechanical strength. For example, the highest increase in compressive strength, 21%, for a dosage of 0.1 wt.% was achieved in [76], while the highest increase, 29%, for a dosage as small as 0.04 wt.% was noted in [58]. For flexural and splitting tensile strength, the relationship seems to be the opposite, with higher dosages resulting in a higher increase: a 0.1 wt.% dosage in [105], with the flexural strength increased by 120%. For the presented optimal dosages of 1.0 wt.%, flexural strength increased by 32% in [33] and compressive strength by 25% in [107], falling between the mean values achieved for lower optimal dosages. It can be concluded that the influence of carbon nanomaterials on flexural and tensile splitting strength is more complex than for compressive strength and can, in some cases, involve additional mechanisms connected to reinforcement-like behaviour. It should be emphasized that these conclusions are derived only from the analysis of optimal dosages and water-to-cement ratios, and the type of nanomaterial utilized for the tested composites can be a crucial factor in their influence on mechanical strength under both compression and tension.

Differences between matrix types tested in the literature were not significant; however, the influence of the interface between aggregates and the matrix can have an effect on the mechanical strength of mortars and concretes. Results acquired for interfaces between matrix and fibres, both steel and polymer, can have an influence on the design of modern multi-scale fibre-reinforced concretes with nanomaterials used to improve the interface area. Further research could seek to test if the interface between traditional rebars and matrices can also be improved with the addition of carbon nanomaterials; however, this would require more complex and costly tests using larger-scale elements. Another path could include the creation of precast elements built fully or partially using cement nanocomposites, which could serve as a strengthening and monitoring layer. Even if precast elements could be created in a more controlled way than that of cast structures, issues related to scaling the production of cement nanocomposites need to be addressed first. In particular, the dispersion of larger dosages and quantities of carbon nanomaterials in a large volume of full-scale elements could prove to be difficult to control.

### 3.6. Electrical Conductivity

Unlike mechanical strength, the very high electrical conductivity of carbon nanomaterials can directly influence the electrical conductivity of the entire cementitious composite. In the absence of a conductive phase, the electrical conductivity of cement-based materials is governed by their humidity and the ionic conductivity of the pore solution [110]. The addition of carbon nanomaterials introduces additional conductive paths, allowing for charge flow with less dependence on humidity. Proper dispersion of carbon nanomaterial in the volume of the composite is crucial for the formation of a continuous network of conductive particles located in close vicinity to each other. This allows for combining ionic conduction with direct conduction through physically connected conductive particles or by quantum tunnelling between them, which does not require physical contact. An important concept in that regard is the percolation threshold, which is often defined as the dosage of the conductive phase, above which further addition has an insignificant influence on the conductivity of the composite. This definition was expanded by Hong [111], who defined the percolation threshold as the “quantity of CNTs for which resistivity changes under varying humidity are negligible”. This crucial difference expands on the definition of the percolation threshold, shifting it towards focusing on the influence of humidity, which, in the case of cement-based composites, is an important factor due to their hygroscopic behaviour and different working conditions. Dosages obtained using this definition can become more practically usable, setting a baseline for composites with low dependence on humidity. In practice, the percolation threshold is mostly dependent on the type of carbon nanomaterial used and its dispersion quality, which means that it can vary largely between studies.

Electrical measurements of cement-based nanocomposites are conducted in various electrode configurations under direct and alternating current. The most popular configurations include two, three and four electrodes, which are embedded in the sample or placed on its surface [112]. Alternating current is often used to eliminate polarization, which takes place under direct current [113]; however, it can also be negated by modifying the measuring setup [114]. Assessment of the electrical properties of cement-based composites can be done using direct measurements that output resistivity values for set parameters of the current or using electrochemical impedance spectroscopy (EIS), which allows for more precise testing of the electrical response of the material on a wider range of frequencies [115,116] and analysing the conductive paths’ contribution to the overall conductivity of the composite [117]. An important conclusion considering measurement techniques was drawn by Pichór [118], who pointed out significant differences in the percolation threshold measured using direct current and EIS.

Literature research on the topic of the electrical conductivity of cement-based nanocomposites focuses mainly on the determination of the influence of various factors and their combinations on the stability and quality of measurements. A crucial influence of humidity is often raised, especially for nanomaterial dosages below the percolation threshold; however, it was noted in [119] that for large CNT dosages, the reduction in humidity reduces the distance between nanomaterial agglomerations and, therefore, improves conductivity. On the other hand, Jang [120] pointed out the importance of reaching the percolation threshold and internal humidity of cement pastes with CNT, emphasizing the balance between conductive paths through ionic conduction and through CNT. The influence of temperature, humidity and the water-to-cement ratio for cement pastes with CNF was tested by Gawel [121]. The influence of temperature was evident through the evaporation of free pore water, which reduced ionic conductivity. A more precise take on the influence of temperature was offered by Wang [122], who tested the electrical conductivity of cement-based nanocomposites with CNF for a temperature range of −30 °C to 100 °C. For a constant 1.0 wt.% CNF, a linear increase in resistivity was observed initially; then, after reaching 20 °C, the relationships were similar to the normal distribution. Besides the influence of temperature and humidity, the porosity of the matrix is believed to influence the electrical conductivity of cement-based nanocomposites. Discontinuations caused by pores [123] and the intrusion of chlorides [124] are some of the factors considered. In the research of Yoo [14], the topic of the influence of the ages of samples of cement pastes with CNT and CNF was considered. An increase in resistivity was observed for all of the samples; however, for CNTs, it was considered negligible.

The main challenges for measuring the electrical conductivity of cement-based nanocomposites are the variety of factors, both internal and external, that can influence readings. Factors such as nanomaterial type and its dispersion quality, the properties of the cement matrix, humidity, temperature, and the influence of measurement methods are difficult to include in combination, and factoring out each of the individual factors might not be possible. The main goal of research conducted in the area of the electrical conductivity of cement-based materials is to implement them as functional materials. Focusing on finding the optimal composition and measurement method to achieve stable and repeatable readings will be decisive for the reliability of these functional materials. Besides challenges arising from the different working conditions of cement-based materials, there is also a need to unify measurement methods in terms of not only the type, amount and materials of electrodes but also the methodology itself [125] since results acquired with different methods can make the interpretation of the influence of other factors difficult.

## 4. Applications of Cement-Based Nanocomposites

The high electrical conductivity conferred to cement-based composites by the addition of carbon nanomaterials is a basis for using them as functional materials. These materials have unique properties and can be used as strain sensors [19,126,127,128,129,130,131,132,133,134,135,136,137,138,139,140,141,142,143,144,145,146], as de-icing surfaces [147,148,149] and for energy harvesting [150,151,152,153,154,155,156,157,158]. These applications make functional materials useful in multiple branches of construction engineering, including monitoring infrastructure, bridges, and civil and industrial structures. Added functionality can improve the safety of use and reduce operational, economic, environmental and retrofitting costs.

### 4.1. Strain Sensors

One of the functional applications of cement-based nanocomposites is as self-sensing materials, which can monitor their own strain and cracks due to differences in or the severance of electrical conductivity. Most researched applications include cement sensors used to measure strains in the volume of the material or attached to existing elements through integration with the matrix [142], on the surface of the element [134], connected via reinforcement [146] or 3D-printed on the surface [144]. Integration with cement matrix is simply submerging small-scale nanocomposite sensors near the surface of the element or placing them on the surface of the tested element directly. Connection with reinforcement allows for the monitoring of strains in the direct vicinity of rebars and also utilizes small-scale nanocomposites. On the other hand, 3D-printing the sensing layer on the surface of the reinforced concrete element allows for the preparation of a uniform and larger sensing surface that is still closely connected to the element. Using cement-based sensors allows for better integration with the measured element and can combine sensing functions with the strengthening or retrofitting of the existing structure.

The main principle for strain measurement used in functional cement-based nanocomposites is piezoresistive behaviour, which describes changes in the electrical resistivity of the material under strain. The effectiveness and sensitivity of the self-sensing material are measured using the related values of fractional changes in resistivity (*FCR*) and gauge factor (*GF*). Fractional change in resistivity is simply a difference in resistivity under assumed levels of strain, while the gauge factor is the relationship of this change to the strain itself. These values can be calculated from measurement results using Equations (1) and (2):

(1) $$FCR=\;\frac{∆R}{R_{0}}*100\%$$

where

*ΔR*—change in resistivity;
*R*_0_—initial resistivity.

(2) $$GF=\frac{FCR}{\varepsilon }\;$$

where

*ε*—strain.

The acquired parameters describe the sensitivity and usability of the sensor. The higher the resistivity change under strain, the higher the sensitivity and, therefore, the ability to detect strains under smaller loads. A comparison of the selected values of *GF* acquired in the literature is given in Table 4.

Testing the piezoresistive properties of cement-based nanocomposites is usually done under cyclical compressive loads in the elastic region of deformation. This regime allows for the observation of reversibility in resistivity changes, which would not be possible under flexural or tensile loads due to the formation of cracks. Therefore, tensile tests are rather rare but have been made for reinforced concrete beams with sensing layers [146]. Some attempts have also been made to use self-sensing cement-based materials as sensors for dynamic effects [136,141,142], which showed a good correlation with results acquired from commercial accelerometers.

Similar to electrical conductivity, with which piezoresistive properties are intrinsically linked, there are multiple factors affecting piezoresistive properties that are considered during testing. The leading factor is humidity, which can vary with the working conditions in which self-sensing materials could be placed. Just like with electrical conductivity, increasing the significance of conduction through nanomaterials might be an answer to this problem. Lu et al. [131] proposed the combined use of micro-scale carbon fibres and fine aggregates covered with graphene to both improve the dispersion of nanomaterials and reduce the composites’ sensitivity to humidity changes. The presence of aggregates can also influence the piezoresistive properties of cement-based nanocomposites. In research by Meoni [136], a less linear and predictable piezoresistive response was measured for concrete samples due to the presence of coarse aggregates. The dispersion quality is yet another important factor required to ensure the proper creation of conductive paths inside the cement matrix and, with it, the quality of its self-sensing ability. One of the solutions to this problem could be covering aggregates with carbon nanomaterials [130,131,137] or synthesizing them directly on cement grains [126,135], concrete additives [127,138] or micro-scale carbon fibres [143]. The influence of the high temperatures characteristic of fire exposure on the piezoresistive properties of functional cement nanocomposites was tested by Dong [139]. After exposure to 300 °C, the sensitivity of sensors was higher due to the partial cleansing of carbon nanotubes from impurities; however, after exposure to 600 °C, a sudden drop in sensitivity was recorded. Similar results were obtained by Nalon [140], who also highlighted the decomposition of the cement matrix at temperatures above 400 °C and an increase in the sensitivity of CNT-based sensors up to 200 °C. The influence of humidity was tested by Carisio [159], who demonstrated the significant influence of humidity on measurements done by sensors made of cement paste with CNT additions. The influence was lower for high dosages of CNT due to the formation of more conductive paths. Tested sensors were unable to measure strain at low humidity; however, they were still able to detect damage that caused severance of electrical conductivity. Similar results were obtained by Tao et al. [160], who added recycled carbon fibres to functional composites. Piezoresistive response depended more on the dosage of the conductive phase rather than the humidity of the composite, which allowed them to sustain a stable gauge factor even at low humidity.

A large amount of research concerning the piezoresistive properties of cement-based nanocomposites has proven a wide interest in functional materials among researchers. A variety of factors influencing the quality of measurements and the sensitivity of the sensor and its reliability encourage the testing of each factor separately and in combination to deepen our understanding of piezoresistive effects, especially in typical working conditions for concrete and reinforced concrete structures. Moreover, future research should cover the practical use of functional materials, considering both material properties and measurement techniques. Laboratory tests on large-scale elements could also bring an important value for practical applications and intensify research towards solving the most pressing issues concerning the production of cement-based nanocomposites on a larger scale. Large-scale tests would also uncover additional mechanisms connected to bigger strain fields and requirements for application techniques.

### 4.2. Heating Materials

Another application of functional cement-based nanocomposites is using their high electrical conductivity to generate heat and use them for de-icing of surfaces. The generation of thermal energy under electrical current is governed by Joule’s heating law (Equation (3)) [161]:

(3) $$P=I^{2}*R$$

where

*P*—power of the system;
*I—*electric current;
*R*—resistance of the system.

Heat-generating materials are used in roads, pavements and bridges to reduce ice accumulation and increase the safety of use. A scaled-up solution using carbon nanotubes and micro-scale carbon fibres was proposed by Nishat in [147]. Heating panels used in this research were made as layered elements consisting of cement nanocomposites, and regular concrete and reinforcement were used as an electrode to provide electricity to the entire composite. A similar solution combining multi-scale carbon materials was proposed by Salim in [149], where a combination of CNTs and micro-scale carbon fibres improved the stability of conductive paths and, with it, heat generation while also improving crack resistance. Besides dispersion quality and nanomaterial dosage, the presence and diameter of the aggregate, as well as the water-to-cement ratio, can be important factors influencing heat generation capabilities [148], similar to functional applications of self-sensing materials. In the case of heat generation, these factors can also impact the thermal conductivity of the composite, as aggregate properties can account for a significant difference in the thermal conductivity of concrete. Just as with other types of functional applications, the constancy of properties with time is important for de-icing applications of cement-based nanocomposites. The addition of silica aerogel was proven in [162] to not only improve the thermal properties of cementitious composites with CNT but also improve the dispersion quality of the nanomaterial and the stability of the conductive paths. In cyclical heating tests, the stability of heat generation was unchanged after 20 cycles. For heating materials to be more economically and environmentally viable, a possibly low voltage can be an important property. In research by Maglogianni [163], high, almost one-hundred-percent efficiency in converting electrical energy into thermal energy was achieved for voltages as low as 2 V. Mortars with CNT and CNF additions were compared, and the best result was acquired for a dosage of 0.1 wt.% CNT, which was explained by the higher thermal conductivity of CNTs themselves.

The durability of functional composites is an important factor for their application as de-icing materials. These materials will be subjected to frequent changes in temperature and corrosive threats from de-icing salts and other chemical sources, such as fuel and chemical spills. Therefore, it is important to not only consider both their durability and integrity as structural materials but also the influence of these degrading factors on functional properties. This topic was undertaken by Yoon et al. [164] for composites with CNT and carbon fibres as the conductive phase. Their results, after 300 cycles, show a significant increase in the resistivity of the composites and a reduction in heating performance by 64%. Conversely, Xu et al. [165] separately investigated the influence of freeze–thaw cycles and chloride attack on the smart properties of cement composites with CNT and found that chloride attack had a negligible influence on the resistivity of samples. More tests on combined effects are required to further refine these results.

Research on the topic of functional cement-based nanocomposites focused on heat generation shows significant progress both in terms of basic properties and possible practical applications. An important issue, just like with other applications of cement-based nanocomposites, is proper dispersion of nanomaterial in the cement matrix to ensure the reliability and even heating of the entire surface. For this kind of functional application to be practically used, an important issue could be related to the technology and viability of covering a large surface with a composite. Another question could be the corrosive resistance of functional composites since roads and pavements are subjected to a multitude of corrosive agents and mechanical wear under cyclical loads.

### 4.3. Energy Harvesting

Energy harvesting from large temperature differences is a dynamically expanding area of research for functional cement-based nanocomposites. It is based on thermoelectric effects that describe the conversion of temperature differences into electrical current and which are widely used in thermocouples, which are temperature sensors used in technological processes. For functional cement materials, a potential application of these effects is seen for facades and roofs, which can be exposed to significant temperature amplitudes. The use of energy-harvesting materials could mitigate the urban heat island effect by converting the excess heat generated into electric energy and reducing the operational costs of buildings [153].

The main effect used in energy harvesting is the Seebeck effect, whose effectiveness can be measured in the value of the Seebeck coefficient. The available literature has explored the use of carbon nanotubes [150,151] and graphene [152,153] for energy-harvesting cementitious composites. In the case of both materials, a stable and high value for the Seebeck coefficient was acquired, along with its good correlation with electrical conductivity, which can potentially predispose these composites to energy-harvesting applications.

Earlier studies frequently concluded that the energy-harvesting mechanism in cement-based nanocomposites was not yet fully understood. However, recent studies published in the last two years indicate substantial progress in this area [154,155,156,157,158]. The research focus has shifted from demonstrating isolated proof-of-concept systems toward understanding the coupling mechanisms governing energy conversion and developing integrated multifunctional materials. Recent reviews suggest that cement-based energy materials are evolving into a broader category, including thermoelectric, piezoelectric, and triboelectric systems integrated with sensing and energy-storage functions. Particularly significant progress has been observed in triboelectric energy-harvesting systems [155,156,157,158]. Recent studies demonstrated integrated self-powered cementitious systems combining triboelectric energy generation with piezoresistive sensing functions for structural health-monitoring applications [166], reaching efficiency in both functionalities. Such systems not only harvest mechanical energy from vibrations or traffic loading but also simultaneously provide sensing capabilities, suggesting a transition from laboratory-scale materials toward intelligent infrastructure applications. Additional advances were reported for conductive cement nanocomposites containing carbon nanomaterials. Recent investigations showed that optimization of conductive networks and interfacial engineering can significantly improve energy conversion efficiency. In triboelectric systems, carbon nanotube-modified cementitious composites were found to provide stable conductive pathways and improved electrical output. Triboelectric nanogenerators based on graphene-reinforced cementitious composites have also proven to be an efficient energy-generating solution, charging a 10 µF capacitor by 3.1 V in one minute [167]. Optimization of carbon nanomaterial content enabled practical electrical outputs sufficient for powering low-energy devices, indicating progress beyond purely conceptual studies. Furthermore, recent thermoelectric studies introduced hybrid CNT-based architectures and dual-mechanism models, accounting for coupled electron and ion transport phenomena. These findings indicate a transition from empirical observations toward physically based explanations of energy conversion mechanisms. Therefore, although a complete understanding of energy harvesting in cementitious nanocomposites remains an open issue, the recent literature suggests that the field has entered a more mature stage, characterized by mechanistic interpretation, multifunctional integration, and practical validation. Consequently, future research should focus less on proving the existence of the effect itself and more on long-term durability, standardization, scalability, and optimization of multifunctional performance.

## 5. Cost-Effectiveness, Sustainability and Environmental Considerations

Practical implementation of carbon-based nanomaterials in cementitious composites requires evaluation not only of material performance but also of economic feasibility, sustainability and environmental impact. Although carbon nanomaterials can substantially improve the mechanical and functional properties of cement-based materials, their practical use remains limited due to relatively high material costs, energy-intensive production methods and difficulties associated with large-scale implementation [168,169].

One of the major economic limitations concerns the cost of nanomaterial production and processing. Additional expenses arise from the need for specialized dispersion procedures, including sonication, surfactants and superplasticizers, required to achieve acceptable nanomaterial distribution in cement matrices. Since the performance of cement nanocomposites strongly depends on dispersion quality, industrial-scale applications may require strict process control and quality assurance systems, further increasing production complexity and cost [168]. Furthermore, recent life-cycle studies indicated that the environmental burden associated with carbon nanotube production itself strongly depends on the manufacturing routes and energy sources used during synthesis [170]. Nevertheless, improved durability, reduced maintenance requirements and multifunctionality may potentially compensate for increased initial costs throughout the service life of structures [171].

From a sustainability perspective, carbon-based nanomaterials may provide indirect environmental benefits through enhancement of material performance and extension of service life. Increased durability and crack resistance may reduce repair frequency and material consumption throughout the life cycle of structures. Furthermore, recent studies suggested that nanomaterials may contribute to decarbonization strategies through performance enhancement and a possible reduction in cement consumption while maintaining the required mechanical properties [172,173]. Multifunctional applications, including self-sensing systems, de-icing pavements and energy-harvesting materials, may additionally contribute to more resource-efficient and intelligent infrastructure systems.

However, environmental impacts associated with cement nanocomposites remain insufficiently investigated. Existing life-cycle studies concerning cement materials focus predominantly on production-related emissions, while ecological consequences associated specifically with nanomaterials are still rarely considered [174]. Previous studies highlighted that environmental aspects associated with nanomaterials remain underrepresented in life-cycle analyses of cementitious materials and require broader consideration [175].

Particular concerns are associated with long-term environmental exposure and end-of-life scenarios. Weathering processes, mechanical degradation and demolition activities may potentially lead to the release of nanomaterial-containing particles into the environment. Carbon nanotubes are known to exhibit high resistance to biodegradation and thermal degradation, creating uncertainties regarding their long-term environmental fate [175]. Potential release of CNT-containing particles from deteriorated cement composites has also been discussed in recent studies, although long-term environmental implications remain insufficiently understood [176].

Recent investigations concerning the environmental toxicity of CNT-reinforced cement mortars demonstrated no significant increase in water toxicity compared with conventional cement-based materials. The observed environmental impact was associated mainly with the leaching of cement hydration products rather than with the direct effects of carbon nanotubes themselves [175]. Similar studies emphasized that the environmental interactions of construction materials occur throughout the whole life cycle and should therefore be evaluated under realistic environmental exposure conditions [175,177].

Therefore, future research should extend beyond optimization of material properties and include comprehensive life-cycle assessment approaches, environmental risk evaluation and standardized methodologies for assessing nanomaterial release and ecological impacts. Such investigations are necessary to ensure safe, economically justified and sustainable implementation of carbon-based cement nanocomposites in future infrastructure systems.

## 6. Conclusions

The addition of carbon nanomaterials influences a wide variety of both basic and functional properties of cementitious composites. This complex influence derives from the intrinsic properties of nanomaterials, mainly their high mechanical strength, hydrophobicity, large specific surface areas and electrical conductivity. Negative effects observed for rheological properties and, in some cases, hydration kinetics can be effectively mitigated through proper composition modifications, optimization of nanomaterial dosage and improvement of dispersion procedures. On the other hand, beneficial effects on mechanical strength, shrinkage behaviour, microstructure refinement and electrical conductivity create opportunities for the use of cement-based nanocomposites as modern multifunctional materials.

The property modifications described in this paper can be summarized as:

- Rheology of fresh mixes—A predominantly negative influence that can be effectively reduced through proper dispersion and composition optimization; this is mainly associated with nanomaterial hydrophobicity, the adsorption of water and the competitive adsorption of superplasticizers.
- Shrinkage—A generally positive influence caused by pore filling, matrix densification and possible water retention effects.
- Hydration heat—No clear consensus exists; positive, negative and negligible influences have been reported due to competing mechanisms, including nucleation effects, water adsorption and interactions with superplasticizers.
- Microstructure—A generally positive influence through the nucleation effect, pore filling and the promotion of hydration products with improved stiffness and matrix densification.
- Mechanical strength—A mainly positive and indirect influence resulting from microstructure refinement and a reduction in porosity, with a smaller contribution from the high strength of carbon nanomaterials themselves.
- Electrical conductivity—A strongly positive influence highly dependent on nanomaterial dosage, conductive network formation and dispersion quality while remaining susceptible to humidity and environmental conditions.

To provide a more balanced and critical analysis of available carbon nanomaterials, comparative tables summarizing their principal characteristics, advantages and limitations were introduced in this manuscript. The comparison demonstrates that no single nanomaterial can currently be considered universally optimal, and the selection of a specific material should depend on the intended application, required functionality and practical implementation constraints. The optimal dosage of the nanomaterial is highly dependent on the desired outcome. For the improvement of basic properties, including mechanical strength, rheological properties and microstructure densification, smaller dosages, up to 0.1–0.2 wt.%, are usually the most beneficial [178]. On the other hand, for the most stable electrical conductivity and functional performance, high dosages of 0.5–1.0 wt.% might be required [178]. Choosing the optimal dosage for both beneficial effects is yet another challenge to overcome for optimizing nanocomposites’ composition.

Functional cement-based nanocomposites represent a rapidly developing research field with the potential to become an important component of future intelligent construction systems. Their multifunctionality and ability to integrate structural and functional roles create opportunities for more efficient and sustainable infrastructure. The multitude of possible applications and compatibility with conventional construction materials make them an attractive area of research and development.

The main benefits associated with the use of these functional materials can be summarized as:

- A potential increase in safety and the expansion of structural health monitoring systems;
- Better integration of sensing systems with structures compared with traditional externally attached sensors;
- Counteracting ice formation and improving the safety of roads, pavements and bridge infrastructure;
- The possibility of energy harvesting and self-powered infrastructure applications;
- A potential reduction in maintenance requirements and the improved durability of structures.

Despite considerable research progress, several important knowledge gaps and limitations remain unresolved. One of the most significant challenges concerns the practical implementation and scalability of cement-based nanocomposites. Current research is still predominantly performed at laboratory scale under highly controlled conditions, while practical implementation in large structural elements may introduce substantial variability.

Major unresolved challenges include:

- Scaling the production of cement-based nanocomposites with regard to production volume, repeatability and nanomaterial dispersion in large-scale applications;
- Development of reliable quality control methods and determination of the influence of combined environmental factors on long-term functionality;
- A better understanding of interactions between nanomaterials, hydration products and chemical admixtures, particularly superplasticizers;
- A more precise assessment of economic and environmental benefits throughout the full life cycle of structures;
- Standardization and unification of testing procedures, measurement techniques and application methods;
- Further investigation of multifunctional mechanisms, including self-sensing, heating and energy-harvesting systems;
- Development of experimental and numerical approaches capable of describing coupled physical and chemical interactions within cement nanocomposites.

Although the economic feasibility of large-scale implementation remains uncertain, the long-term operational, environmental and functional benefits may outweigh the increased production costs. Future research should therefore focus not only on improving individual material properties but also on reliability, standardization and practical applicability under realistic operating conditions. Such developments will be necessary for the transition of cement-based nanocomposites from laboratory materials to practical engineering solutions.

## Figures and Tables

**Table 1:** Selected rheological parameters of cement-based nanocomposites with carbon nanomaterials available in the literature. Under “dosage”, the dosage with the strongest influence is given.

Matrix	Nanomaterial	Dosage	w/c	Type of Test	Change	Ref.
Paste	CNT	0.15 wt.%	0.55	Yield stress	+270%	[56]
Paste	CNT	0.5 wt.%	0.18	Yield stress	+51 times	[57]
Paste	GO	0.04 wt.%	0.42	Yield stress	−82%	[58]
Paste	GO	0.15 wt.%	0.80	Yield stress	+146%	[59]
Paste	GO	0.06 wt.%	0.35	Yield stress	+12 times	[60]
Paste	GO	0.07 wt.%	0.40	Yield stress	+441%	[61]
Mortar	CNT	0.2 wt.%	0.50	Yield stress	+506%	[62]
Concrete	CNT	10 wt.%	0.48	Yield stress	−84%	[63]

**Table 2:** Selected results of shrinkage tests of cement-based nanocomposites with carbon nanomaterials available in the literature. Under “dosage”, the dosage with the strongest influence is given.

Matrix	Nanomaterial	Dosage	w/c	Type of Shrinkage	Change	Time	Ref.
Paste	GO	0.05 wt.%	0.42	Chemical	−25%	28 d	[67]
Paste	GO	0.03 wt.%	0.50	Chemical	−4%	672 h	[68]
Paste	CNT	0.6 wt.%	0.44	Chemical	−30%	7 d	[66]
Paste	CNT	0.1 wt.%	0.40	Autogenous	−43.6%	150 h	[73]
Paste	CNT	0.05 wt.%	0.30	Autogenous	−22.1%	28 d	[71]
Mortar	CNT	0.1 wt.%	0.45	Drying	−62%	7 d	[74]
Concrete	CNT	0.1 wt.%	0.55	Autogenous	−54%	7 d	[76]
Concrete	GO	0.08 wt.%	0.44	Drying	+7.45%	60 d	[79]
UHPC	CNF	0.15 wt.%	0.20	Autogenous	+20%	28 d	[80]

**Table 3:** Selected results of mechanical strength tests of cement-based nanocomposites with carbon nanomaterials available in the literature. Under “dosage”, the optimal dosage of the carbon nanomaterial is given, as concluded by the authors of the cited study.

Matrix	Nanomaterial	Dosage	w/c	Type of Mechanical Strength	Increment	Curing Time	Ref.
Paste	fCNT	0.05 wt.%	0.40	Compressive	13.80%	28 days	[47]
Paste	GNP	0.1 wt.%	0.48	Compressive	10.00%	28 days	[27]
Paste	GO	0.04 wt.%	0.42	Compressive	29.00%	7 days	[58]
Paste	GO + SiO_2_	0.01 wt.%	0.40	Compressive	27.17%	28 days	[61]
Paste	CNT	0.075 wt.%	0.40	Flexural	49.89%	28 days	[51]
Paste	CNT	0.08 wt.%	0.50	Flexural	25.00%	28 days	[38]
Paste	CNT + GNP	0.05 wt.% CNT + 0.025 wt.% GNP	0.40	Flexural	78.80%	28 days	[32]
Paste	GO	0.03 wt.%	0.40	Flexural	66.56%	28 days	[29]
Paste	GO + SiO_2_	0.02 wt.%	0.38	Flexural	26.00% 31.00%	7 days 28 days	[39]
Paste	CNT	0.15 wt.%	0.40	Splitting	50.00%	7 days	[82]
Mortar	fCNT	0.1 wt.%	0.45	Compressive	6.00%	28 days	[48]
Mortar	CNF	0.02 wt.%	0.35	Compressive	24.00%	14 days	[49]
Mortar	fCNF	1.0 wt.%	0.50	Compressive	25.00%	28 days	[107]
Mortar	CNT	0.05 wt.%	0.35	Compressive Flexural	23.00% 29.00%	28 days	[74]
Mortar	fMWCNT	0.1 wt.%	0.50	Flexural	120.00%	28 days	[105]
Mortar	Graphene	0.05 wt.%	0.50	Tensile	79.00%	28 days	[108]
Mortar	MWCNT	1.0 wt.%	0.50	Splitting	32.00%	7 days	[33]
Concrete	CNT	0.1 wt.%	0.55	Compressive	21.00%	28 days	[76]
Concrete	GNP	0.025 wt.%	0.50	Compressive	17.00%	28 days	[36]
Concrete	GO	0.08 wt.%	0.35	Compressive	12.65%	28 days	[79]
UHPC	CNF	0.1 wt.%	0.18	Compressive	18.00%	28 days	[109]
UHPC	CNF	0.3 wt.%	0.20	Tensile	55.00%	28 days	[80]

**Table 4:** Selected results of gauge factor values acquired in the literature.

Matrix	Nanomaterial	Dosage	GF	Type of Test	Measurement Type	Ref.
Mortar	CNT	1 wt.%	25.36	Dynamic	Embedded	[142]
Paste	CNT	0.25 wt.%	748	Compressive	Bulk	[126]
Mortar	CNT	2 wt.%	6544	Compressive	Bulk	[127]
Paste	CNT	1 wt.%	63.30	Compressive	Bulk	[128]
Paste	GNP	1 wt.%	100	Compressive	Bulk	[132]
Mortar	CNF	1.2 wt.%	1552	Compressive	Bulk	[133]
Paste	CNF	5 wt.%	78.4	Compressive	On surface	[134]
Mortar	CNF	0.2 wt.%	18.7	Compressive	Bulk	[135]
Concrete	CNT	1 wt.%	20	Dynamic	Bulk	[136]
Paste	CNT	0.5 wt.%	168	Compressive	Bulk	[139]
Paste	fCNT	0.5 wt.%	244.88	Compressive	Bulk	[141]
Paste	CNT	0.5 wt.%	451	Compressive	Bulk	[145]
Mortar	CNT	0.8 wt.%	972.87	Flexural	On rebars	[146]

## Data Availability

No new data were created or analyzed in this study. Data sharing is not applicable to this article.
